# Survey of European clinical geneticists on awareness, experiences and attitudes towards direct-to-consumer genetic testing

**DOI:** 10.1186/gm449

**Published:** 2013-05-22

**Authors:** Heidi Carmen Howard, Pascal Borry

**Affiliations:** 1 INSERM, UMR 1027, Epidemiology and Public Health, Faculté de médecine Purpan, Université Paul Sabatier, 37 allées Jules Guesde, 31000, Toulouse, France; 2 Department of Public Health and Primary Care, KU Leuven, Kapucijnenvoer 35 Box 7001, 3000 Leuven, Belgium

## Abstract

**Background:**

The advent of direct-to-consumer (DTC) genetic testing (GT) has sparked a number of debates regarding the scientific validity of tests, their broad health and ethical implications for society as well as their legal status. To date, relatively few empirical studies have been published regarding this phenomenon. We conducted a survey of European clinical geneticists to gauge their awareness of, experiences with, and attitudes towards DTC GT.

**Methods:**

We invited 300 clinical geneticists from 28 European countries to complete an online questionnaire. Statistical analyses of closed-ended questions were performed using the STATISTICA software package. Answers to open-ended questions were analysed for recurring themes.

**Results:**

One hundred and thirty-one clinical geneticists answered our survey (response rate, 44%). Eighty-six percent (110/128) of respondents were aware of DTC GT, and over one-third had been contacted by at least one patient regarding these services. The majority (84%) of respondents did not agree with telephone medical supervision outside of an established doctor-patient relationship. The majority of clinical geneticists also found it unacceptable to provide non-face-to-face medical supervision for: (i) a presymptomatic test for a condition with very high penetrance; (ii) a predictive test for a condition that has a 'medium' penetrance of 50% to 60%; and (iii) carrier testing. For conditions that are neither treatable nor preventable and for disorders with serious health consequences, clinical geneticists were almost unanimous in expressing the unacceptability of offering such genetic tests outside of the traditional healthcare setting, without an established physician-patient relationship and without face-to-face medical supervision.

**Conclusion:**

A high percentage of European clinical geneticists are aware of DTC GT and the majority do not agree with the model of provision used by many commercial companies for certain severe or actionable health conditions. Despite this disagreement with the DTC model of provision, >85% of respondents said that they would offer genetic counselling to patients who asked for a consultation after having undergone DTC genetic testing. The understanding of the views and opinions of this expert stakeholder group should be considered in the attempts to shape responsible policy and guidelines for these services.

## Background

Direct-to-consumer (DTC) genetic testing (GT) involves the advertising and/or the offer of genetic testing directly to consumers outside of the traditional healthcare system. Since 2006, there has been an increasing number of private companies, many of which are based in the USA, selling such services. Companies offer genetic tests for both monogenic disorders as well as common complex diseases. Other tests provide information regarding pharmacogenomics, ancestry, paternity, fetal sex determination, and non-disease traits such as eye or hair colour. Although many companies sell genetic tests to consumers without the intermediary of a healthcare professional, some companies have recently turned to a new model of DTC genetic testing wherein a healthcare professional is involved [1]. Given the fact that prior to this commercial offer of genetic testing services, the usual route to obtaining a genetic test was through a consultation with a medical professional within the traditional healthcare system, it may not be surprising that this new source of test provision has fuelled a number of debates between many different stakeholders including, among others, medical doctors, researchers, lawyers, policy makers, patient advocates and ethicists [2-4]. Nonetheless, to date, relatively little empirical data has been published regarding the views of different stakeholder groups. A number of studies have looked at the views of the general public (including studies whereby individuals purchased genetic testing as part of the research project) [5-7] while a few articles have reported the views of actual users of DTC genetic testing services [8-10]. Indeed, these articles report primarily on publics and users from the USA except for that by Wilde *et al*. (2010) which was conducted in Australia [7], and the study by Su *et al*. (2011) which was conducted using Internet blogs without specifying the geographic location [10]. Only limited information is available regarding the awareness and the utilization of DTC genetic testing services by the general public in Europe. A study in the United Kingdom among 4,050 twins who volunteered in the TwinsUK Adult Twin Registry showed that only 13% had heard of 'personal genome testing' and that 5% were very or fairly likely to order a test if the cost was £250 [11]. Furthermore, a study conducted in Greece reported that the vast majority (82.1%) of respondents from the general public were 'against' DTC GT, and of these, most wanted a physician to refer them to GT services and to explain the test results to them [12].

A small number of studies have looked at the views of healthcare professionals regarding DTC GT (for a review please see Goldsmith *et al*., 2013) [13] Recently, a number of publications have reported primarily on the views of genetic counsellors regarding this phenomenon. [14-16] Hock *et al*. gathered data in 2008 on the knowledge and beliefs of 312 American genetic counsellors, almost half of whom had at least one patient initiate a discussion on DTC GT in the last 2 years and 14% had been asked to discuss or interpret DTC genetic test results[16]. Giovanni and co-authors (2010) reported primarily on the experiences of 22 genetic test providers (91% of whom were genetic counsellors) in the USA who had provided consultations to consumers who had purchased DTC GT [15]. These 22 providers who performed a DTC GT consultation represent 16.5% of the 133 providers who completed the study questionnaire [15]. Brett *et al. *[14] recently reported on the experiences of 130 genetic counsellors and 38 clinical geneticists (members of the Human Genetics Society of Australasia); they focused primarily on the description of 25 clients who had consulted 19 healthcare professionals regarding DTC GT. Six empirical articles focusing primarily on physicians' views of, and/or experiences with DTC GT have been published to date. Ohata and colleagues [17] studied the views of 1,145 general practitioners, and 294 clinical geneticists in Japan. Kolor and colleagues [18] briefly reported on the survey of healthcare professionals with different specialties (that is, family physicians, internists, paediatricians, dermatologists). Meanwhile, Powell and colleagues [19,20] published two articles regarding the survey responses of 382 family and internal medicine providers from one state (North Carolina) in the USA. They reported not only on awareness, opinions and experiences but also on the educational needs of these physicians with respect to DTC GT [19]. Bernhardt and colleagues [21] also report on a survey of the experiences and views of 502 family medicine and internal medicine physicians in the USA. Finally, Haga and co-authors (2011) studied the attitudes and use of 157 physicians who are part of a national network of primary care physicians in the USA (MDVIP), which in December 2008, announced a collaboration with Navigenics [22].

Information specifically regarding the views of North American physicians specialized in clinical geneticists has not yet been published in a full-length article. However, a survey of members of the American Society of Human Genetics (ASHG) was presented at the ASHG annual meeting (Montreal, Canada, 2011) by Wicklund *et al. *[23] regarding the views of ASHG members' towards the regulation of DTC GT services The authors reported that two-thirds of the 1,517 American survey respondents (67.7%, *n *= 1,027) 'agreed or strongly agreed that the involvement of a healthcare provider should be required when using DTC genetic tests' [23]. Moreover, the authors revealed that a clear majority (93.6%) of the respondents agreed or strongly agreed that 'regulation of DTC genetic tests is important, and 51.9% disagreed or strongly disagreed that current regulations are sufficient'. We have published our data on the question of banning certain types of DTC genetic tests in a separate publication [24]. Similarly, we found that a majority of respondents strongly or somewhat agreed (69% and 62%, respectively) with banning genetic tests sold DTC for prenatal gender tests and for genome scans.

Although a number of guidelines have been published by different national bioethics societies in Europe (such as, Switzerland, France, Portugal and Austria) as well as various national organisations and the European Society of Human Genetics, to date, there is only one published article presenting limited empirical data regarding the views of European healthcare professionals in Greece. In this publication the focus is genetics in general and the questions about DTC GT are only one part of the questionnaire [12]. In order to begin to fill this gap, we conducted a survey of clinical geneticists in Europe. Herein, we present empirical evidence, both quantitative and qualitative, regarding the awareness of, experiences with, and attitudes towards DTC GT services of clinical geneticists in Europe.

## Materials and methods

### Identification of clinical geneticists

With the help of the websites of national European genetic associations, national contact people, the website Orphanet, and the results of previous studies [25-27], a list of clinical institutes where medical genetic consultation is offered to patients was compiled. Institutes from 28 European countries were included; in total, 300 institutes were identified. From these institutes, the email address and contact address of (mostly) the head of the institutes were gathered. All 300 institutes were contacted with the aim of receiving one completed questionnaire per institute. Medically qualified specialists in genetics who have offered genetic counselling to patients in the last year were asked to complete a survey of items assessing their awareness of, experience with, and attitudes towards DTC GT. The data collection took place between May and December 2010. In total, five email reminders were sent out in the attempt to increase the response rate. No monetary or other incentive was offered to participants. The response rate was calculated based on the number of partially and completely filled questionnaires we received from different individuals divided by 300.

### Questionnaire

DTC GT was defined in the letter of invitation as 'genetic tests and test interpretations sold directly to consumers rather than through the traditional model whereby a health care provider must be consulted before a test can be performed'. We elaborated a 35-item questionnaire in English specifically for this study. The questions were based on ethical and social issues related to DTC GT identified in the literature. We posed 28 closed-ended questions (the majority of which were answered on a five-point Likert scale) and seven open-ended questions asking respondents to elaborate on their closed-ended answers. In total we posed six questions regarding basic characteristics, eight regarding experiences (including awareness) and 21 regarding attitudes towards DTC GT (full questionnaire available upon request). We reported on six of the latter group of questions regarding the subject of banning certain types of DTC genetic tests in a previous publication [24]. We report on the remaining 15 questions pertaining to attitudes as well as seven of the questions regarding experiences herein. Since DTC genetic testing services encompasses a large variety of different tests and models, instead of basing our questions solely on the definition of, or simply the words 'DTC genetic testing' our aim was to be more specific by focusing our questions on particular aspects that differentiate the DTC GT context from testing within the traditional clinical setting (that is, lack of established patient-doctor relationship, consultation by telephone, services offered outside the traditional healthcare system). Before being sent to clinical geneticists for the study, this questionnaire was reviewed by 10 experts from various backgrounds (clinical genetics, genetic counselling, ethics and social sciences) and was adapted based on their comments. The questionnaire was distributed and filled out online by respondents using an Internet-based survey tool called surveygizmo.

### Statistical analysis

Statistical analyses were performed using the STATISTICA software package (Version 9, 2009). Descriptive statistics were calculated for all closed-ended questions. Under the assumption that lacking data are missing completely at random, missing values were excluded from analyses. Differences between two independent groups were calculated using the Mann-Whitney U test; differences between 3 or more independent groups were calculated using Kruskal-Wallis Anova. Differences between dependent groups were calculated using Wilcoxon matched pairs test. Given the multiple tests performed in this study, a more conservative *P *value ≤0.001 is considered significant herein.

Based on the countries where clinicians practice, respondents were grouped based on geographical location (that is, North, East, South, West). Geographical regions were based on those described by the United Nations [28]. The number of hours clinicians spend counselling patients each week was also grouped into groups: 10 h or less, 11-20 h, and >21 h. Answers to open-ended questions were read and analysed for recurring themes. This study conforms to the Declaration of Helsinki and was approved by the Medical Ethics Commission of the Faculty of Medicine at KU Leuven.

## Results

### Characteristics of respondents

A total of 131 fully or partially filled questionnaires were returned from respondents in 28 countries, resulting in a response rate of 44% (131/300). Half of the clinical geneticists who responded to our survey were men (66/131). (Table 1) The average year of birth was 1957 (SD, 8.1; range, 1941 to 1983) with 48% and 32% of respondents born during the 1950s and 1960s, respectively. The average number of years of practice in their capacity as healthcare professionals in clinical genetics who regularly offer consultations with patients regarding genetic issues is 21 years (SD, 8.3; range, 2 to 40 years). The majority of respondents (45%, 59/131) spend between 11 h and 20 h per week in consultation with patients regarding genetics issues; 34% (44/129) spend <11 h per week and 21% (27/129) spend >21 h. Different European regions were equally represented and 46.5% of respondents work in five of the 28 countries surveyed.

**Table 1:** Characteristics of survey respondents

Characteristic	Respondents
Female	49.6%

	

*Patient consultation per week (h)*	

1-10	34%

11-20	45%

>20	21%

*Geographic location *	

North	25%

East	18%

West	28%

South	29%

*Countries with highest percentage of respondents*	

Italy	11.5%

UK	11%

Germany	9%

Spain	8%

France	7%

Average year of birth	1957

Average years in practice	21

### Awareness

Eighty-six percent (110/128) of the clinical geneticists expressed that they are aware that companies are advertising and selling genetic tests directly to consumers. No significant differences in awareness (or any of the other answers) were observed based on geographic location of respondents, their sex, birth year (or birth decade), hours offering counselling to patients per week, or years in medical practice.

Of the 110 physicians who are aware of DTC GT services, 64% (70/110) were able to name at least one company. When asked to list up to five companies offering such tests, a total of 67 distinct company names were mentioned (Table 2). The existence of each company mentioned and the exact model of provision were not verified. The top three most cited companies were 23andme, deCODE (which sells the DTC service deCODEme) and Navigenics.

**Table 2:** List of companies named by clinicians when they were asked to name DTC GT companies.

Company name	Times cited (*n*)
23andme	43

deCODE	21

Navigenics	12

Gendia	8

Myriad	7

Counsyl	6

GHC	5

Sciona	4

Knome	3

The table only includes companies that were named three or more times.

### Experience

Thirty-four percent (42/124) of the respondents (Table 3) have been contacted by at least one patient who addressed the DTC GT subject but had not (yet) purchased or taken a DTC GT test. Of these clinicians, the majority (52%, 22/42) had been contacted by one to five patients, and 26% (11/42) had been contacted by six to 20 patients who addressed the topic of DTC GT. Interestingly, 12% (5/42) of this group of respondents saw >100 patients in this context. The majority of clinicians wrote that patients asked about the quality and the relevance of the tests. Specifically, some of the topics were related to notions of 'clinical significance', 'validity', 'usefulness', 'accuracy of tests', 'medical relevance' and 'benefits'. Some clinicians were also approached with questions about specific types of testing: pharmacogenomics, paternity, ancestry and disease testing (that is, breast cancer, prostate cancer). Other patients asked basic practical information regarding price and availability of tests.

**Table 3:** Clinicians who have had patients consult them about DTC genetic testing.

Patients seen who have asked about DTC GT (*n*)	Clinicians who have seen patients who have asked about DTC GT but had not (yet) purchased a test (*n*)	Clinicians who have seen patients who have asked about DTC GT after having purchasing a test (*n*)
0	82	67

1-5	22	35

6-10	6	6

11-20	5	6

21-50	2	2

51-100	2	1

100+	5	4

Total	124	121

Forty-four percent (54/121) (Table 3) of the respondents have had at least one patient contact them after having undergone a DTC GT. The majority of these (65%, 35/54) have been contacted by between one and five patients in such a situation. Most of the 25 geneticists who expanded on this answer stated that patients wanted to have results interpreted and explained. For example, one clinician wrote that 'They [the patients] did not know how to interpret the results and were confused about it' (Respondent 16).

Very few clinicians were able to name the companies used by their patients; seven clinicians named at least one company, with a total of 23 company citations. The company most often cited to have been used by patients was 23andme (4/23) followed by deCODE, GHC and Myriad which were all cited twice.

### Attitudes

#### Replacement of a face-to-face consultation with a telephone consultation

Eighty-four percent of respondents strongly or somewhat disagreed with replacing face-to-face medical supervision by a medical doctor with telephone supervision by a medical doctor outside of the context of an established doctor-patient relationship (Table 4). Moreover, none of the respondents strongly agreed with this situation. For the same question placed within the context of an established doctor-patient relationship, opinions were more evenly divided, with 41% of respondents somewhat or strongly agreeing with the replacement of face-to-face supervision with telephone supervision. In general, many respondents agreed with telephone medical genetic supervision only under certain conditions: (a) if they knew the patient well; and/or (b) if the patient lived far away; and/or (c) if the patient had reduced mobility; and/or (d) if the telephone conversation would encourage a face-to-face consultation. Examples of comments made by respondents on this subject include:

**Table 4:** Clinical geneticists' attitudes regarding the replacement of a face-to-face consultation with a telephone consultation within and outside of the context of an established doctor-patient relationship.

Scenario/Type of test	Strongly disagree	Somewhat disagree	Neither agree or disagree	Somewhat agree	Strongly agree
**WITHOUT **the context of an established physician-patient relationship, it is acceptable to replace a face-to-face medical genetic supervision (by a medical doctor) with telephone supervision (by a medical doctor)	63% (74/117)	21% (24/117)	5% (6/117)	11% (13/117)	0/117 (0%)
**WITHIN **the context of an established physician-patient relationship, it is acceptable to replace face-to-face medical genetic supervision (by a medical doctor) with telephone supervision (by a medical doctor)?	25% (29/118)	27% (32/118)	8% (9/118)	33% (39/118)	8% (9/118)

'I see no reason not to answer by phone or mail when I know the patient. However, serious matters will only be discussed within the frame of a consultation. In cancer-prone families, I often use the phone to establish a first contact with people requiring presymptomatic testing that do not come spontaneously to the genetic consultation. This first contact is only meant as an incentive to come to a consultation and does not replace a consultation.' (Respondent 5)

'If the physician-patient relationship has been formally established, I think that, in some instances, it could be possible to discuss by phone if the patient lives far away from the hospital, and/or needs some more explanations about a genetic result that has been discussed face-to-face.' (Respondent 38)

'In a face to face conversation there is of course room for words but for reactions, attitudes and mimics analysis too. This proximity is essential to establish relations with the proband. Nevertheless, in a well-established relationship with (of course well identified) a well-known subject, phone call may help to facilitate a continuity as a milestones between face to face appointments. While this could be affordable during follow-up of a patient this must not replace an essential human contact.' (Respondent 36)

#### No face-to-face supervision

Table 5 presents the clinical geneticists' opinions regarding the acceptability of providing particular types of genetic tests without a face-to-face medical consultation. The majority (87%, 86% and 60%) of clinical geneticists considered it totally unacceptable to provide medical supervision that is not face-to-face for the following situations: (i) a presymptomatic test for a condition with very high penetrance; (ii) a predictive test for a condition that has a 'medium' penetrance of 50% to 60% and for carrier testing, respectively. The results of the Wilcoxon-Matched pairs test show that respondents consider it less unacceptable to provide supervision without a face-to-face consultation for carrier test information and for low-risk genes than for presymptomatic tests with a high penetrance (98% to 100%) (Z = 4.56, *P *= 5 × 10^-6^; Z = 4.60, *P *= 4 × 10^-6^) or 'medium' penetrance (Z = 4.94, *P *= 1 × 10^-6^; Z = 5.22, *P *<1 × 10^-6^). This being said, only 11% and 16% of respondents thought it was somewhat or totally acceptable to offer testing for a gene with very low relative risk and for carrier testing, respectively, without face-to-face medical consultation.

**Table 5:** Acceptability of providing genetic tests without face-to-face medical supervision.

Scenario/Type of test	Totally unacceptable	Slightly unacceptable	Neutral	Slightly acceptable	Totally acceptable
A presymptomatic test that can predict if an asymptomatic person has a very high probability (98-100% penetrance) of developing a condition	87% (103/119)	3% (4/119)	3% (4/119)	3% (3/119)	4% (5/119)
A predictive test for a condition that has a penetrance of 50% to 60%	86% (100/118)	7% (8/118)	3% (3/118)	4% (5/118)	2% (2/118)
A predictive test for a condition that increases or decreases a person's risk of developing this disease by 4% when compared to the general population (that is, relative risk of 1.04 and lifetime risk of 1.4%)	47% (55/119)	25% (30/119)	18% (21/119)	7% (8/119)	4% (5/119)
A carrier test for homozygous monogenic disorders	60% (71/119)	19% (23/119)	5% (6/119)	6% (7/119)	10% (12/119)

Physicians were asked to consider 'For the following situations, let us know if you think it is acceptable to provide genetic tests **without face-to-face medical supervision **by choosing the number (option) that best corresponds to your opinion'.

Many respondents mentioned the need for genetic counselling before and/or after GT in order to make sure that patients understand results and their consequences.

'Genetic testing must be preceded by genetic counseling. If not, the whole issue can give you more troubles than benefits.' (Respondent 105)

'Testing should be accompanied by explanation and counselling because the client must be aware of the limitations of the test, interpretation of the results, and the consequences of the result.' (Respondent 126)

'I believe that any genetic test without one or more sessions of pretesting counselling followed by post testing counselling is totally unnacceptable.' (Respondent 81)

The importance of context and the type of testing being done was also raised as being essential to consider:

'Serious personal risks should have face-to-face medical supervision. I could leave less serious on the responsibility of the consumer, who wants the test.' (Respondent 30)

#### No established physician-patient relationship and no face-to-face consultation

When asked about their attitudes towards the provision of different types of genetic tests outside of the traditional healthcare system without an established physician-patient relationship and without a face-to-face consultation, clinical geneticists were almost unanimous in expressing the unacceptability of such testing for conditions that are neither treatable nor preventable and for disorders with serious health consequences (such as, neurological impairment) (Table 6). Moreover, no respondent strongly agreed in either of these categories. For conditions where preventive or therapeutic measures can be offered, fewer physicians strongly disagreed (70%), however including those that 'disagree somewhat', nonetheless, brings the total percentage of those who disagree to 90%. Finally, for traits or conditions with no or relatively mild health repercussions, 74% strongly or somewhat disagreed with the offer under the above mentioned circumstances.

**Table 6:** Physicians' views regarding the acceptability of providing different types of genetic tests outside of the traditional healthcare setting, without an established physician-patient relationship and without a face-to-face consultation.

Type of genetic test	Strongly disagree	Somewhat disagree	Neither agree or disagree	Somewhat agree	Strongly agree
When preventive or therapeutic measures can be taken based on genetic test results, it is acceptable to offer a genetic test without face-to-face medical supervision	70% (80/114)	20% (23/114)	3% (3/114)	6% (7/114)	1% (1/114)
For conditions that are neither treatable nor preventable it is acceptable to offer a genetic test without face-to-face medical supervision	94% (106/113)	4% (4/113)	2% (2/113)	1% (1/113)	0% (0/113)
For conditions with serious health repercussions (such as neurological impairment) it is acceptable to offer a genetic test without face-to-face medical supervision	97% (110/114)	2% (2/114)	1% (1/114)	1% (1/114)	0% (0/114)
For traits or conditions that have either no or relatively mild health repercussions (such as ear lobe shape or gluten insensitivity) it is acceptable to offer a genetic test without face-to-face medical supervision	39% (44/114)	35% (40/114)	10% (11/114)	14% (16/114)	3% (3/114)
					

Physicians were asked to consider 'The following statements are set in a situation outside of the traditional health care system whereby there is no established physician patient relationship. Please choose the option that best represents your agreement with each statement.'

#### Attitudes towards providing genetic counselling

When asked 'Would you provide counselling to a patient who comes to see you with a test result for a presymptomatic test obtained through a direct-to-consumer genetic testing company?' 86% (97/113) said yes, and 14% (16/113) said no. Of those who said yes, 68% (66/97) elaborated on their answer. A large majority stated they would offer counselling because it was their duty to help patients. Comments include:

'A physician's role is to assist a patient whatever the circumstances.' (Respondent 36)

'Genetic counselling must be provided no matter how the test is done.' (Respondent 105)

Other respondents mentioned that offering counselling would minimise any harm from having purchased DTC genetic tests.

'It's like treating the injuries of a drunk driver after an accident - it should not have happened, but now you have to minimise the damage.' (Respondent 44)

Of those who said they would not offer counselling reasons for not offering counselling included not wanting to 'support' the companies' activities as well as a lack of resources (time, money). One respondent mentioned that he/she did not want to take 'responsibility':

'I am not willing to take such responsibility. It should be very difficult and dangerous task - I am willing to participate only in the complete testing process - from the first explanation to the result interpretation.' (Respondent 22)

## Discussion

We present herein the first results of a questionnaire study regarding the awareness, experiences and views of European clinical geneticists about DTC GT. Moreover, this is, to our knowledge, the only study that specifically focuses on the views of clinical geneticists. Comparison with other published surveys' results of healthcare professionals on this topic [14-17,19-22] is not very informative due, in part, to the differences in target audiences, and also due to the fact that the actual nature of the questions posed differs a great deal. In our questionnaire, we focused primarily on posing questions based on the specific characteristics of the DTC model of providing GT which make it different from the traditional model of test provision. In particular, we addressed the lack of an established physician-patient relationship, the lack of face-to-face consultation and the offer of genetic tests outside of the traditional healthcare system. Given the great heterogeneity between companies, posing such specific questions allowed us to obtain more detailed information than that acquired from questions referring more generally to the term 'direct-to-consumer genetic testing'.

Our results reveal that clinical geneticists value face-to-face genetic counselling over telephone counselling, however, they do accept the latter under certain conditions within the context of an established physician-patient relationship. Admittedly, in-person genetic counselling represents the traditional and presently most widely used model of providing counselling in a clinical setting. However, other models of provision of counselling are also being incorporated into clinical genetics, including telephone, group and telegenic counselling (via video conference or web-link, which can also be considered face-to-face) [28]. Indeed, the lack of appropriate counselling and provision of information within the DTC GT context is a serious concern [14-17,19,20,22,29,30].

Our data also show that European clinical geneticists have a nuanced view with regards to the different types of traits and diseases for which DTC GT are offered. For example, offering a consultation that is not face-to-face was less acceptable to clinicians for conditions with high penetrance than for low penetrance conditions or carrier testing. This is in line with the recommendations for genetic counselling related to GT as developed in the context of the Eurogentest project [31].

Furthermore, outside of the traditional healthcare system, without a face-to-face consultation, and without an established doctor-patient relationship, almost all clinicians surveyed strongly disagreed with the offer of genetic tests for conditions with serious health repercussions but less than half disagreed with offering genetic tests for conditions with limited health repercussions. This supports the notion of not addressing all different types of DTC GT in the same way. In the report 'A Common Framework of Principals for direct-to-consumer genetic testing services' (2010), the Human Genetics Commission also categorizes genetic tests into different groups, and then specifically makes recommendations for some groups and not others [32]. Some authors [33] have also suggested that not all DTC GT be treated equally when it comes to attempts to regulate them and address the social and medical needs of consumers. How feasible this is in practice remains to be seen.

Interestingly, our data also offer information regarding how well-known or established different companies are within the European clinical geneticist population. Consequently, this could be a sign of how effective different companies' advertising efforts have been. When we asked clinicians to recall which companies were used by the patients who had purchased tests, few clinicians could recall the companies used, nonetheless, those who could recall the names, most frequently expressed that patients had purchased genetic tests from 23andme, deCODE, GHC and Myriad. Along the same line, when we asked clinicians which companies they could name 'off the top of their heads' the companies most often mentioned by geneticists were 23andme, deCODE and Navigenics. Brett *et al*. reported that almost half of the 25 patients who purchased DTC GT had purchased the services from 23andme or Counsyl [14] while Giovanni and co-authors reported that 50% of the testing was purchased from four companies: 23andMe (22.7%), Navigenics (9.1%), DNADirect (9.1%) and Genelex (9.1%) [15]. These results suggest that these are, indeed, the companies that have most successfully advertised their services to consumers as well as to healthcare professionals in the USA, Australia and many European countries.

Our results also reveal that there may be some confusion about the exact meaning of DTC GT. A number of respondents mentioned the names of companies that are not strictly speaking, based on information present on their websites, considered DTC GT companies (that is, not offering nor advertising DTC). An alternate explanation may be that some companies are not necessarily presenting all their services on their websites (that is, not explicitly stating that they sell DTC but will sell tests when presented with requests from consumers). Either way, if this confusion can be generalized to clinical geneticists and counsellors, it could undermine some of the responses of surveys that simply use the term 'direct-to-consumer' GT in their questionnaires without providing a definition for the term.

From our survey, 86% (110/128) of European clinical geneticists reported being aware of DTC GT while 68% (198/291) of clinical geneticists in a Japanese study stated they were aware of DTC GT [17]. The higher level of awareness of our respondents may be due to the fact that Ohata and colleagues conducted their survey three years prior to ours (September and October 2007), at a time that can be considered relatively early in the development of the DTC GT phenomenon. They also posed the question specifically about awareness of particular types of DTC genetic tests: 'DTC genetic testing kits for the classification of predisposition to obesity or the prediction of susceptibility to hypertension/diabetes mellitus/osteoporosis have become commercially available. Do you know about the sale of such test kits?' Therefore, answering in the negative would not necessarily mean not knowing about DTC genetic tests in general. Forty-two percent of the 1880 DocStyles respondents (*n*=790) were aware of DTC GT services and this difference in awareness may be also due to the fact that this survey was conducted in 2008 but also perhaps due to the fact that none of the healthcare professionals involved in the survey were genetics specialists *per se*. The increasing awareness among healthcare providers is in line with increasing awareness in the general population [34].

As mentioned by Brett *et al*. (2012), the size of the DTC GT market and therefore its impact on clinical services is difficult to assess. Based on our study, 42% and 54% of respondents had at least one patient contact them regarding DTC genetic testing without having purchased and after having purchased testing services respectively. Six percent of the clinical geneticists who responded to Ohata and colleagues survey reported having a consultation with a patient regarding DTC GT [17]. Hock *et al*. report that 46% of genetic counsellors (146/312) had worked with patients who brought up DTC GT and 14% had patients who wanted to address their DTC GT results [16]. While Giovanni *et al. *[15] and Brett *et al. *[14] reported that 16.5% (22/133), and 11.3% (19/168), respectively, stated having at least one patient consult them regarding the results from DTC GT companies. About 6% (*n*= 118) of the respondents of the DocStyles survey reported having at least one patient bringing in DCT GT results to discuss in the past 12 months [18]. Again, our higher percentage may simply be due to the fact that we collected data later, except that the Australian study took place months after ours. The above results are also in line with studies that measured the impact of DTC advertising for hereditary breast cancer testing on genetic services which showed an increase in patient interest in the test as well as an increase in the number of genetic tests ordered by medical professionals [35-37]. Furthermore, five and four of the respondents in our study reported to have been contacted by over 100 patients regarding DTC GT before and after having purchased testing, respectively (Table 3). This is a very high number and we speculate whether this is (partly) due to a confusion on the part of these physicians regarding the exact meaning of DTC GT. Alternately, some countries may have companies that are advertising (if not also selling) some tests DTC and this has resulted in the high number of consultations. This issue is particularly important when trying to assess how much of the resources from the public or traditional healthcare system will be consumed by customers of DTC GT companies in the form of referrals to physicians within the public system.

### Limitations

The response rate of 44% (131/300) is somewhat less than the mean response rate of 56% calculated in a review of surveys conducted with healthcare professionals [38]. The corresponding 131 returned questionnaires did not allow for the power needed to detect subtle differences in subcatergories (that is, differences in responses based on years in practice or based on regional location). Furthermore, we are aware that those clinicians who did respond to our survey may be geneticists who have particularly stronger (negative) opinions towards DTC GT and/or have had more experience with patients requesting information about these services than non-respondents. In this way, our results may not be generalizable to all European clinical geneticists and additional studies looking at non-responders' characteristics should be performed. Moreover, we sent invitations mostly to the directors of clinical genetics institutes whom are likely to be older, and have practised clinical genetics under more traditional models of testing and counselling and therefore may not be as open to newer models. That being said, the average year of birth of respondents is 1957 (SD, 8.1; range, 1941 to 1983) and the average years in practice is 21 years (SD, 8.3; range, 2 to 40 years) hence there is still a good range of years of experience of respondents represented in this study.

## Conclusions

This questionnaire study is the first to report on the views, attitudes and experiences of European clinical geneticists regarding DTC GT. As experts in clinical genetics, it is essential to obtain the opinion of this stakeholder group and include these views in the ongoing discussion about DTC GT services.

A pertinent question is how such empirical research can contribute to policy making around DTC GT. This question is inherently related to other more fundamental questions: What role should be attributed to empirical research in bioethical debates? How do empirical research findings implicitly embody certain norms and values? In what way can results from empirical work help resolve ethical dilemmas [39]? The classical distinction between empirical and normative approaches, however, has been challenged and more dynamic interactions between both approaches have been fostered. Majority opinions do not necessarily lead to ethical normative conclusions. This would consist of a logical fallacy. However, quantitative surveys such as ours, play a role in understanding the extent to which the phenomenon of DTC is present in the current practice of clinical geneticists; it helps us understand the experiences and views of experts who, on a daily basis, deal with patients with heritable disorders and their families; it helps us understand the motivations, reasons and argumentations that support their views. This supports the view that normative and descriptive approaches complement each other: 'Good studies in normative ethics will be grounded in good empirical data. Good descriptive studies will be shaped by ethical theory, providing a framework in which the data will be interpreted. Ethical reflection is enhanced when these two types of investigation are undertaken in an interdisciplinary and cooperative fashion' [40].

Based on the results of our questionnaire study, a few concrete points may be particularly relevant for policy makers. Firstly, the results show that a number of clinical geneticists in Europe have already been confronted with patients who have undergone a DTC genetic test. Although this study was not designed to measure the impact of DTC GT on the healthcare system, our results show that a downstream impact on the healthcare system may be expected. Considering that most DTC genetic tests currently provided lack of clinical utility, this potential cascade effect should be kept in mind by policy makers as it could lead healthcare professionals to devote more time and energy to clinical activities that are not medical priorities. This supports the plea for more regulatory control over the quality of the genetic tests presently being offered to consumers. As stated by the European Society of Human Genetics statement on DTC GT for health purposes, 'In order to prevent premature translation of genomic services into the market or clinical practice, a regulatory oversight will be required. Oversight will be important to synthesize available evidence on the clinical validity and utility of emerging genetic tests and to identify current gaps in knowledge, as well as the studies and measures needed to resolve them' [41]. Similar statements for more regulatory control (in general) have also been advanced by various governmental and professional organizations [42,43]. In a previous study [44], we described how different European countries have developed diverging regulatory approaches to genetic testing for health purposes which apply to DTC genetic tests. Various countries (such as France, Portugal or Switzerland) have stipulated in their legislations that genetic tests can only be carried out by a medical doctor after the provision of sufficient information concerning the nature, meaning and consequences of a genetic test and after the consent of the person concerned. France has introduced a penalization of users in relation to DTC GT [45]. In the Netherlands, to be offered DTC, some genetic tests need a permit from the Minister of Health before they can be offered to the public [46]. Moreover, other countries may have a regulatory framework that could apply to DTC GT, but the interpretation and eventual application of that framework may not be clear. In that regard, it is not surprising that many stakeholders may consider the regulatory framework insufficient in their countries and ask for increased regulatory control.

Furthermore, it is important to emphasize that based on our results of the experiences of clinical geneticists with patients who have purchased DTC GT services, it is clear that various consumers want their genomic test results to be interpreted and explained by healthcare professionals. Moreover, ordering a genetic test outside of the traditional healthcare system does not appear to be considered an impediment for consumers to return to their physicians (within the traditional healthcare system) with these results. Most of the clinical geneticists who responded were willing to counsel such patients based on their medical duty. Further effort, time and funding should, therefore, be placed specifically on studying the potential downstream impact of DTC GT on the healthcare system.

Second, clinical geneticists are not eager to replace face-to-face consultation with a telephone consultation. Only in very specific situations (for example, if they know the patient well; if the patient lives far away; or if the telephone conversation would encourage a face-to-face consultation) would they be willing to consider this. Moreover, most respondents considered it totally unacceptable to provide medical supervision that is not face-to-face in situations such as a presymptomatic test for a condition with a very high penetrance or a predictive test for a condition that has a 'medium' penetrance of 50% to 60%. These results are relevant in the elaboration of best practice guidelines for telecounselling and genetic counselling. They raise questions regarding which type of circumstances allow for the highest quality of genetic counselling.

Third, most clinicial geneticists mentioned the need for genetic counselling before and/or after GT. These results highlight concerns these professionals have with regard to the provision of genetic tests without adequate medical supervision and appropriate information provision. As mentioned above, these views should be taken into account when formulating best practices and guidelines for information provision. As it stands, there appears to be a double standard between what is expected from clinicians who offer GT in the traditional clinical setting *versus *what is accepted of DTC companies. It is important that stakeholders in genetics and genomics, including researchers, physicians, consumers, patients and policy makers, address these differences in standards to avoid confusion and potential erosion of standards meant to assure that patients obtain adequate and ethical GT services. When addressing this issue, it will be important to keep clinicians' views and preferences in mind as well as conduct further research to obtain the views and preferences of other stakeholders such as consumers, patients and hospital administrators

## List of abbreviations

DTC: direct-to-consumer; GT: genetic testing

## Competing interests

The authors declare that they have no competing interests.

## Authors' contributions

HCH and PB both conceived and designed the study and wrote the manuscript. PB was responsible for administering the survey and data acquisition. HCH was responsible for data analysis. All authors read and approved the final manuscript.
